# Synthesis of kinase inhibitors containing a pentafluorosulfanyl moiety†
†Electronic supplementary information (ESI) available. CCDC 154150–154153. For ESI and crystallographic data in CIF or other electronic format see DOI: 10.1039/c7ob02289a


**DOI:** 10.1039/c7ob02289a

**Published:** 2017-09-29

**Authors:** Supojjanee Sansook, Cory A. Ocasio, Iain J. Day, Graham J. Tizzard, Simon J. Coles, Oleg Fedorov, James M. Bennett, Jonathan M. Elkins, John Spencer

**Affiliations:** a Dept of Chemistry , School of Life Sciences , University of Sussex , Falmer , BN1 9QJ , UK . Email: j.spencer@sussex.ac.uk; b UK National Crystallography Service , Chemistry , University of Southampton , Highfield , Southampton , SO17 1BJ , UK; c Structural Genomics Consortium , Nuffield Department of Clinical Medicine , University of Oxford , Oxford , OX3 7DQ , UK; d Structural Genomics Consortium , Universidade Estadual de Campinas , Campinas , SP 13083-886 , Brazil

## Abstract

A series of 3-methylidene-1*H*-indol-2(3*H*)-ones substituted with a 5- or 6-pentafluorosulfanyl group has been synthesized by a Knoevenagel condensation reaction of SF_5_-substituted oxindoles with a range of aldehydes. The resulting products were characterized by X-ray crystallography studies and were tested for biological activity *versus* a panel of cell lines and protein kinases. Some exhibited single digit nM activity.

## Abstract 2

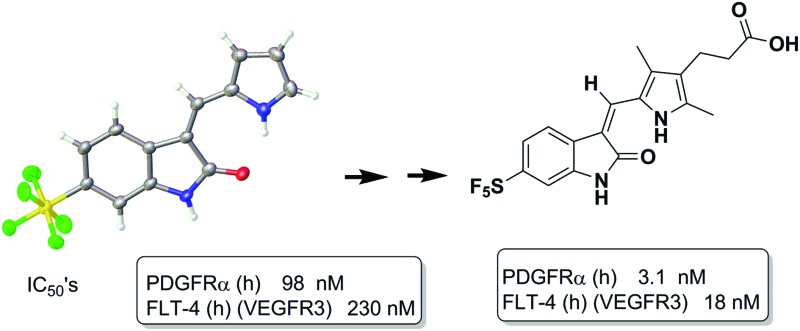
A series of 3-methylidene-1*H*-indol-2(3*H*)-ones substituted with a 5- or 6-pentafluorosulfanyl group has been synthesized by a Knoevenagel condensation reaction of SF_5_-substituted oxindoles with a range of aldehydes.

## Introduction

**Fig. 1:**
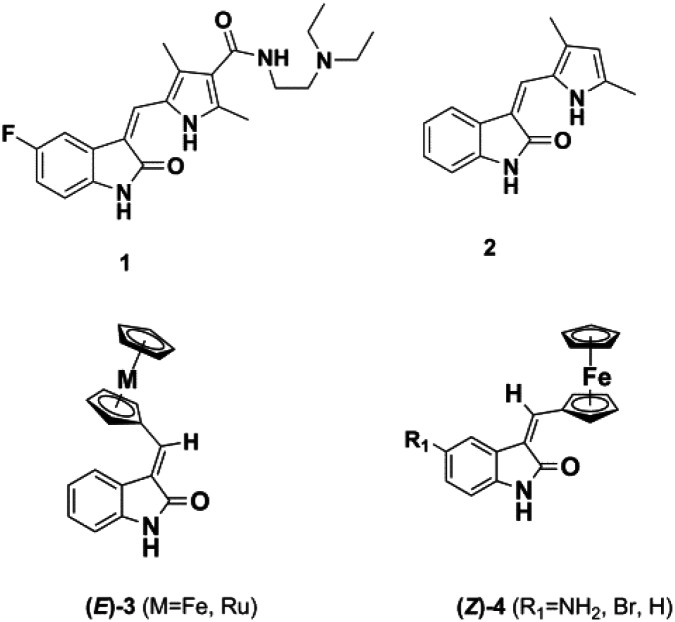
Oxindole-based kinase inhibitors.

The dysregulation of protein phosphorylation mediated by protein kinases is key to the progression of a number of cancers. Unsurprisingly, a number of ATP-competitive kinase inhibitors are in clinical use and development.^1^–^7^ For example, the oxindole-containing antiangiogenic drug Sunitinib **1**, containing a 5-fluorine substituent and a solubilizing side chain on the pyrrole unit, is in clinical use and superseded Semaxanib (**2**, SU5416) (Fig. 1) as well as inspiring a number of other studies on druglike oxindoles.^8^–^15^

Metal-based analogues such as **3**, **4** have been described by our group and show kinase inhibition down to the nM range and tolerance of a range of substituents at the C-5 position.^16^,^17^

**Fig. 2:**
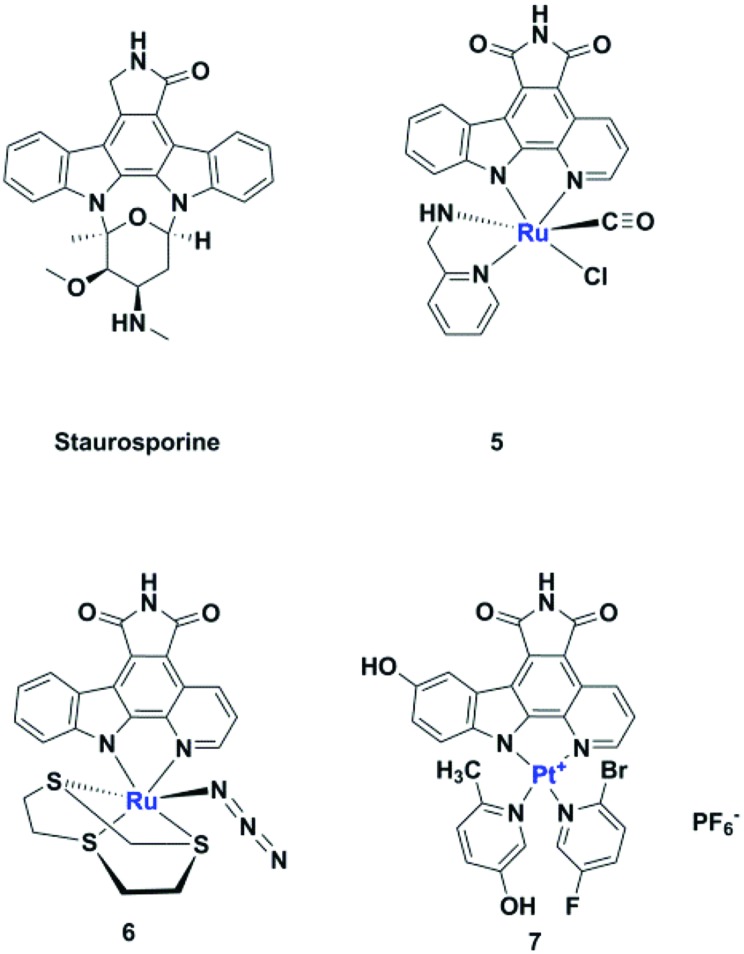
Staurosporine analogues.

Meggers's group replaced the sugar unit in staurosporine, a pan-kinase inhibitor with relatively high toxicity and unsuitable for clinical use, by square planar and octahedral transition metal complexes **5–7**, leading to highly potent, selective kinase inhibitors. This was attributed to the novel “imaginary hypervalent carbon” geometry enabled by the metal complexes (Fig. 2, **5–7**).^18^–^21^

The pentafluorosulfanyl group is attracting increasing interest in medicinal chemistry. Displaying strong polarity, high lipophilicity and good stability under physiological conditions, an SF_5_ substituent has often been shown to behave like a CF_3_ group.^22^–^26^ Here we show that a SF_5_ group can be incorporated in both classical and metal-based oxindole derivatives, at the 5- or 6-position, leading to analogues displaying kinase inhibition down to the nM range.

## Results and discussion

**Scheme 1:**
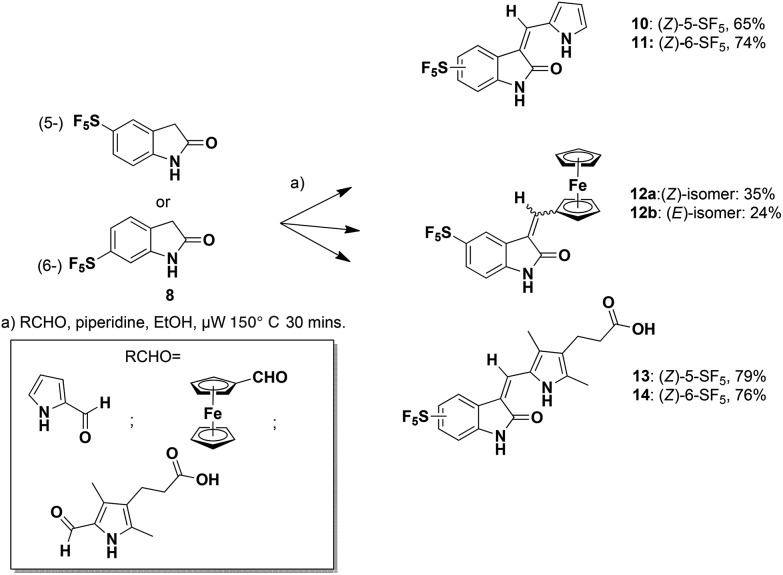
Microwave-mediated Knoevenagel condensations.

Microwave-mediated Knoevenagel condensations of the commercially-available 5- or 6-SF_5_-substituted oxindoles **8** 27 with three separate aldehydes led to the products **10–14** (Scheme 1).^28^

**Fig. 3:**
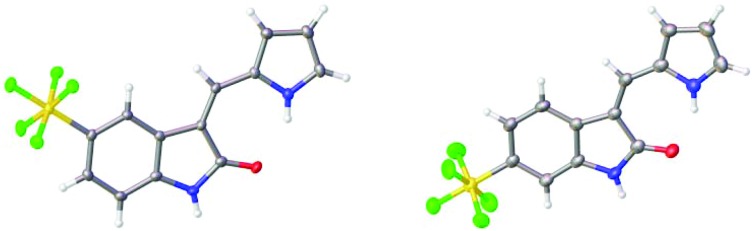
Solid state structures of **10** and **11**.

The structures of the pyrrole-containing positional isomers **10** and **11** were confirmed by ^1^H NMR, ^13^C NMR spectroscopy, elemental analysis and mass spectrometry. In their ^1^H NMR spectra the most downfield signals were assigned to the pyrrole-NH groups (*δ* 11.10–13.40 ppm) due to an intramolecular NH···O=C hydrogen bond and further confirmation of their anticipated *Z*-configuration and such a hydrogen bond was provided in the solid state (Fig. 3).^29^

**Fig. 4:**
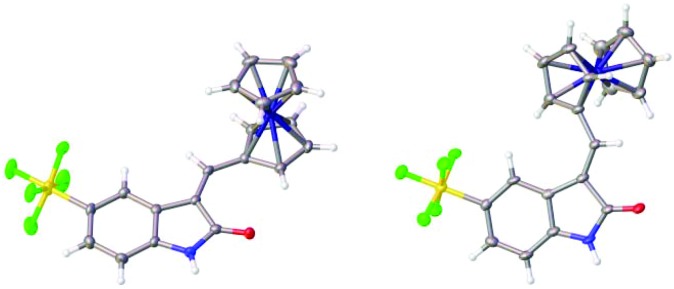
Solid state structures of **12a** and **12b**.

The related reaction with ferrocene carboxaldehyde afforded a mixture of stereoisomers **12a** and **12b**, which were separated by chromatography. Both isomers were characterized in the solid state (Fig. 4).

We tested all synthetic compounds against a panel of kinases in a biochemical assay. Each data point was measured in duplicate (technical replicates). The potencies of compounds that showed appreciable (approx. 50%) inhibition at 1 μM concentration were established by testing them over a dose range to determine their IC_50_ values. Additional kinase binding studies were performed *vs*. a select group of functionally and structurally divergent kinases including AAK1 (Adaptor-associated protein kinase 1), BMP2K (BMP-2-inducible protein kinase, where BMP is bone morphogenic protein), GAK (Cyclin G-associated kinase) and STK16 (Serine/threonine-protein kinase 16) (Table 1). In all assays a control of staurosporine, a known promiscuous kinase inhibitor, was used.

**Table 1:** Biochemical kinase assays

		Kinase^*a*^	**10**	**11**	**12a**	**12b**	**13**	**14**	Staurosporine^*c*^
1	IC_50_ (M)	STK16^*b*^	1.76 × 10^–5^	1.35 × 10^–4^	nt	nt	—	—	1.14 × 10^–7^
2		GAK^*b*^	3.42 × 10^–5^	4.76 × 10^–7^	nt	nt	—	—	1.89 × 10^–8^
3		BMP2K^*b*^	4.52 × 10^–7^	1.87 × 10^–4^	nt	nt	—	—	3.17 × 10^–9^
4		AAK1^*b*^	1.0 × 10^–6^	1.0 × 10^–3^	nt	nt	—	—	2.47 × 10^–9^
5^*d*^		DYRK3 (h)	—	—	1.7 × 10^–6^	2.4 × 10^–6^	—	—	4.5 × 10^–8^
6		PDGFRα (h)	—	9.8 × 10^–8^	—	—	—	3.1 × 10^–9^	1.2 × 10^–9^
7		FLT-4 (h) (VEGFR3)	—	2.3 × 10^–7^	—	—	5.3 × 10^–7^	1.8 × 10^–8^	7.8 × 10^–10^

^*a*^Unless stated otherwise, performed in the presence of 10 μM ATP.

^*b*^Binding displacement assays have no ATP present.

^*c*^No activity was observed for **10–14***vs.* KDR kinase (h) (VEGFR2), PDGFRβ kinase (h); DYRK1a (h); DYRK2a (h); FLT-1 kinase (h) (VEGFR1), where staurosporine positive controls gave IC_50_s of 2.3 × 10^–9^; 2.5 × 10^–9^; 3.2 × 10^–8^; 8.3 × 10^–7^; 2.8 × 10^–8^ respectively.

^*d*^Entries 5–7 performed by CEREP (France; http://www.cerep.fr). nt – not tested. — insufficiently active for an IC_50_ determination.

In the case of a number of kinases, *e.g.* VEGFR2 (vascular endothelial growth factor receptor 2) and DYRK2 (Dual-specificity tyrosine phosphorylation-regulated kinase 2), no appreciable inhibition was observed for any of our synthesized compounds, suggesting that we might observe differences in their selectivity, *i.e.* no promiscuity, towards this panel of kinases. Compound **10** bound to BMP2K with an IC_50_ of 452 nM whereas **11** displayed nM potency *vs.* PDGFR2 (98 nM) and submicromolar potency *vs*. VEGFR3 (230 nM). Stereoisomeric **12a** and **12b** only inhibited DYRK3 in the low micromolar range. The positional isomers **13** and **14** both inhibited VEGFR3 with IC_50_s of 530 and 18 nM respectively whereas the latter displayed an excellent 3.1 nM IC_50_*vs*. PDGFRα.

The synthesized compounds were next tested in breast cancer and non-transformed breast cell lines. Compounds **10** and **11** potently inhibited MCF7 and T47D breast cancer cell proliferation with GC_50_ values ranging from 0.35 to 3.8 μM with compound **11** proving superior to compound **10**.

MCF7 and T47D cells are luminal A ER^+^/PR^+^/HER2^–^ cells that would normally be responsive to estrogen and progesterone receptor (ER/PR) antagonists such as tamoxifen and megestrol respectively, but not to human epidermal growth factor receptor 2 (HER2) inhibitors. MDA-MB-231 (abbreviated as MM231) cells are triple negative (ER^–^/PR^–^/HER2^–^) and cannot be treated with hormone receptor and EGFR (HER2) inhibitors, making cancer cells such as these refractory to most treatment strategies. Compounds **10** and **11** may offer advantages for the treatment of ER^+^/PR^+^ cancer cells by polypharmacologically targeting multiple kinases such as the receptor tyrosine kinases and other serine/threonine kinases. Lastly, it is encouraging that normal MCF10A cells were resistant to all inhibitor treatments suggesting these compounds would have a large therapeutic window (Table 2).

**Table 2:** Cellular activity of **10** and **11**

		GC_50_ ^*a*^, μM		
Compound	MCF7	T47D	MDA-MB-231	MCF10A
**10**	4.8 ± 1	0.49 ± 0.4	na	na
**11**	0.69 ± 0.4	0.35 ± 0.1	na	na

^*a*^The GC_50_ value was defined as the amount of compound that caused 50% reduction in cellular proliferation in comparison with DMSO-treated control and was calculated using GraphPad Prism version 6 software; na = not applicable.

Compound **11**, which bears a methylidene indolinone scaffold (Fig. 1), demonstrated its greatest potency against the receptor tyrosine kinase PDGFRα, which adopts an inactive conformation according to X-ray crystallographic analysis (Fig. S1B†); however, an X-ray co-crystal structure containing a methylidene indolinone-based inhibitor (**15**, Fig S1†) bound to the RET kinase domain reveals a type 1 inhibitor binding-mode, or binding to an active kinase conformation (Fig. S1B†). Alignment of **15**-bound RET with the PDGFRα structure reveals gross structural shifts between analogous β-hairpins and Cα-helices, which is not surprising as the active conformation is generally rigid and condensed and the inactive conformation is generally more open.^30^ Alignment of the Dasatinib-bound co-crystal structure of Protein-tyrosine kinase 6 (PTK6), a non-receptor tyrosine kinase, with the **15**-bound RET reveals that they share a similar, active conformation (Fig. S1C†). Based on this analysis, it makes sense to use an active kinase conformation, as the above elements (β-hairpin and Cα-helix) are proximal to the ATP-binding pocket and likely to have an impact on binding mode. However, rather than performing docking studies with RET, we decided that PTK6 would be superior as this kinase has a threonine gatekeeper residue, similar to that of PDGFRα, whereas RET has a valine at the same position. Valine is slightly bigger and more hydrophobic than threonine, lacking a hydroxyl group compared to threonine, and could drastically perturb interactions necessary for **10** and **11**-binding. Furthermore, based on the similarity of **10** and **11** with other type 1 methylidene indolinone inhibitors, we predicted that docking these compounds to an active PTK6 kinase conformation would yield improved binding energies; a result confirmed by docking **10** and **11** to the inactive kinase conformation of PDGFRα (PDB: ; 5K5X), which reported higher binding energies, and thus less avid binding, for both **10** and **11**.

**Fig. 5:**
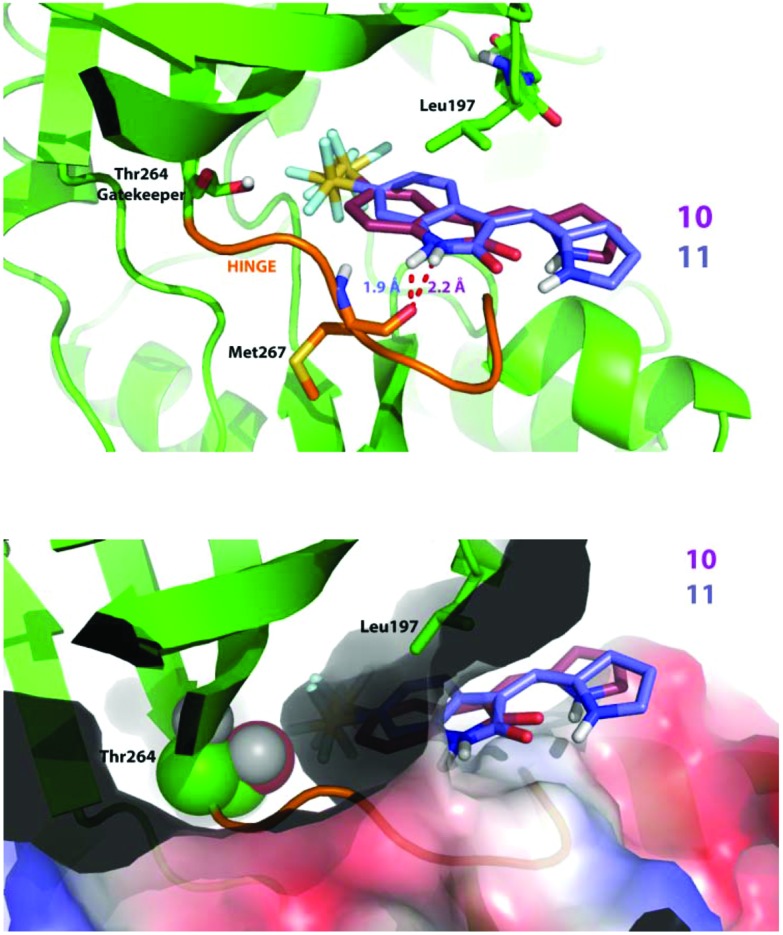
Docking poses of **10** and **11**. Docking was performed using AutoDock 4.2.6.; Lamarckian Genetic Algorithm empirical free energy scoring function. PDB format files for the ligand and kinase domain were pre-processed using AutoDock Tools 1.5.6.

Against PTK6, both compounds bind in a very similar manner as seen in Fig. 5 (top panel). We found the SF_5_ moiety of **10** and **11** to bind deeply in a predominantly hydrophobic pocket next to the gatekeeper residue (Fig. 5 top and bottom panels). The amide hydrogen of both compounds interacts with the Met267 backbone; however, note that the attachment of the SF_5_ group to position 5 of the oxindole ring forces compound **10** to swing away slightly from the hinge. This may explain why inhibitor **11** is more potent in cells and *in vitro* (PDGFRα & VEGFR3) as the hydrogen bond distance is shorter for the **11** docking-pose, indicative of a stronger interaction.

## Conclusion

A small library of SF_5_-containing oxindole analogues has been synthesized. Many products were characterized in the solid state and assayed *vs.* a small panel of kinases. Docking studies predicted effective binding of the SF_5_ group to a hydrophobic cleft in the kinase and biochemical assays showed little evidence of promiscuity in the range of analogues synthesized. This bodes well for the use of the SF_5_ group in medicinal chemistry with compound **14** in particular showing low nM potency against VEGFR3 and PDGFRα kinases.

## Experimental

5-(Pentafluorosulfanyl)-1,3-dihydro-indol-2-one and 6-(pentafluorosulfanyl)-1,3-dihydro-indol-2-one were obtained from SpiroChem (https://spirochem.com/sf5.html). Ferrocene carboxaldehyde, pyrrole-2-carboxaldehyde and piperidine were obtained from Sigma-Aldrich. Preparative TLC plates were obtained from Analtech. Solvents and reagents were purchased from commercial suppliers and were used without purification. All reactions were performed in a fume hood. NMR spectra were recorded on Varian 500 MHz or 400 MHz spectrometers and chemical shifts are reported in ppm, usually referenced to TMS as an internal standard. LCMS were performed by Shimadzu LCMS-2020 equipped with a Gemini® 5 μm C18 110 Å column and percentage purities were ran over 30 minutes in water/acetonitrile with 0.1% formic acid (5 min at 5%, 5%–95% over 20 min, 5 min at 95%) with the UV detector at 254 nm. Mass spectrometry: ESI mass spectra were obtained using a Bruker Daltonics Apex III, using Apollo ESI as the ESI source. For EI mass spectra, a Fissions VG Autospec instrument was used at 70 eV. Analyses are for the molecular ion peak [M]^+^ and are given in *m*/*z*, mass to charge ratio. Elemental analyses were conducted by Stephen Boyer (London Metropolitan University). A CEM Explorer microwave unit was used for microwave reactions (under fumehood) with the hood placed down. The following CCDCs have been deposited for the solid-state structures presented herein: **10** = ; 154150; **11** = ; 154151; **12a** = ; 154152; **12b** = ; 154153.†

### (*Z*)-3-(1*H*-Pyrrol-2-yl)methylene-5-pentafluorosulfanylindoline-2-one, **10**

5-(Pentafluorosulfanyl)-1,3-dihydro-indol-2-one (129.6 mg, 0.5 mmol), pyrrole-2-carboxaldehyde (57.06 mg, 0.6 mmol), ethanol (5 mL) and cat. piperidine (3 drops) were subjected to microwave irradiation by ramping to 150 °C and were held at that temperature for 30 minutes. TLC analysis of the cooled reaction mixture monitored consumption of starting materials. The crude reaction mixture was extracted with ethyl acetate (2 × 10 cm^3^) and washed with deionised water (10 mL) and brine (2 × 10 mL), the organic layer was dried using magnesium sulphate then filtered through a cotton wool plug. The crude mixture was concentrated *in vacuo* and purified using silica gel column chromatography using 3 : 7 hexane/diethyl ether to give an orange solid. The yield was 105 mg, 65%. Crystallization by mixed solvents, CH_2_Cl_2_ and hexane, provided orange crystals. ^1^H NMR (DMSO-d_6_, 500 MHz): *δ* = 13.22 (1H, s, NH), 11.30 (1H, s, NH), 8.24 (1H, d, *J* = 2.3 Hz, CH), 8.11 (1H, s, CH), 7.65 (1H, dd, *J* = 8.6, 2.2 Hz, CH), 7.44 (1H, d, *J* = 2.2 Hz, CH), 7.02 (1H, d, *J* = 8.6 Hz, CH), 6.92 (1H, d, *J* = 3.6 Hz, CH), 6.41 (1H, dd, *J* = 3.6, 2.2 Hz, CH). ^13^C NMR (DMSO-d_6_, 126 MHz): *δ* = 169.9, 147.5, 141.5, 130.0, 129.5, 127.6, 125.9, 124.7, 122.5, 116.7, 115.2, 112.3, 109.6. HRMS-ESI (*m*/*z*) found: 337.0431, calc. for [C_13_H_9_F_5_N_2_OS + H]^+^ 337.0429. Anal. calcd (%) for C_13_H_9_F_5_N_2_OS: C, 46.43; H, 2.70; N, 8.33; found (%): C, 46.55; H, 2.61; N, 8.21.

### (*Z*)-3-(1*H*-Pyrrol-2-yl)methylene-6-pentafluorosulfanylindoline-2-one, **11**

6-(Pentafluorosulfanyl)-1,3-dihydro-indol-2-one (129.6 mg, 0.5 mmol), pyrrole-2-carboxaldehyde (57.06 mg, 0.6 mmol), ethanol (5 mL) and cat. piperidine (3 drops) were subjected to microwave irradiation by ramping to 150 °C and were held at that temperature for 30 minutes. TLC analysis of the cooled reaction mixture showed consumption of starting materials. The crude reaction mixture was extracted with ethyl acetate (2 × 10 mL) and washed with deionised water (10 mL) and brine (2 × 10 mL), the organic layer was dried using magnesium sulphate then filtered through a cotton wool plug. The crude mixture was concentrated *in vacuo* and purified using silica gel column chromatography using 3 : 7 hexane/ethyl acetate and trituration with hexane to give brown-orange solid. The yield was 142 mg, 74%. Crystallization in CH_2_Cl_2_ (DCM) provided orange crystals. ^1^H NMR (DMSO-d_6_, 500 MHz): *δ* = 13.31 (1H, s, NH), 11.14 (1H, s, NH), 7.99 (1H, s, CH), 7.81 (1H, d, *J* = 8.6 Hz, CH), 7.53 (1H, dd, *J* = 8.6, 2.0 Hz, CH), 7.48 (1H, s, CH), 7.26 (1H, d, *J* = 2.0 Hz, CH), 6.93 (1H, m, CH), 6.43 (1H, dd, *J* = 3.7, 2.1 Hz, CH). ^13^C NMR (DMSO-d_6_, 126 MHz): *δ* = 169.5, 138.8, 130.2, 130.0, 129.6, 128.3, 123.1, 119.1, 118.7, 114.7, 112.7, 107.0. HRMS-ESI (*m*/*z*) found: 337.0432, calc. for [C_13_H_9_F_5_N_2_OS + H]^+^ 337.0429. Anal. calcd (%) for C_13_H_9_F_5_N_2_OS: C, 46.43; H, 2.70; N, 8.33. Found (%): C, 46.59; H, 2.61; N, 8.17.

### 5-Pentafluorosulfanyl-3-ferrocenylindolin-2-one, **12a,b**

5-(Pentafluorosulfanyl)-1,3-dihydro-indol-2-one (259.2 mg, 1.0 mmol), ferrocenecarboxaldehyde (256.8 mg, 1.2 mmol), ethanol (10 mL) and cat. piperidine (6 drops) were subjected to microwave irradiation and work-up as above. The crude mixture was concentrated *in vacuo* and purified using preparative TLC using 3 : 7 hexane/ethyl acetate to give fraction 1 (purple solid; 160 mg, 35%) and fraction 2 (red solid; 109 mg, 24%). Crystallization of fraction 1 was by mixed solvents (CH_2_Cl_2_ and hexane) and fraction 2 was by CH_2_Cl_2_ alone. (***Z***)-**12a**. ^1^H NMR (DMSO-d6, 500 MHz): *δ* = 10.84 (1H, s, NH), 8.23 (1H, s, CH), 7.98 (1H, s, CH), 7.68 (1H, d, *J* = 8.6, CH), 6.92 (1H, d, *J* = 8.6 Hz, CH), 5.37 (2H, s, 2CH), 4.69 (2H, s, 2CH), 4.22 (5H, s, Cp). ^13^C NMR (CDCl_3_-d, 126 MHz): *δ* = 167.7, 141.9, 125.1, 119.3, 116.0, 110.0, 108.4, 74.0, 73.3, 70.0, 60.3, 14.2. HRMS-ESI (*m*/*z*) found: 455.0065, calc. for [C_19_H_14_F_5_FeNOS]^+^ 455.0060. Anal. calcd (%) for C_19_H_14_F_5_FeNOS: C, 50.13; H, 3.10; N, 3.08. Found (%): C, 50.22; H, 3.03; N, 3.07. (***E***)-**12b**. ^1^H NMR (DMSO-d6, 500 MHz): *δ* = 10.94 (1H, s, NH), 8.30 (1H, s, CH), 7.76(1H, d, *J* = 8.4, CH), 7.65–7.71 (1H, m, CH), 7.01 (1H, d, *J* = 8.4, CH), 4.79–7.81 (4H, m, 4CH), 4.29 (5H, m, Cp). ^13^C NMR (CDCl_3_-d, 126 MHz): *δ* = 171.1, 141.8, 109.0, 88.2, 72.6, 71.7, 70.2, 60.3, 31.5, 29.6, 22.6, 20.9, 19.0, 14.1, 14.0. HRMS-ESI (*m*/*z*) found: 455.0064, calc. for [C_19_H_14_F_5_FeNOS]^+^ 455.0060. Anal. calcd (%) for C_19_H_14_F_5_FeNOS: C, 50.13; H, 3.10; N, 3.08. Found (%): C, 50.27; H, 3.23; N, 3.10.

### (*Z*)-3-(2,4-Dimethyl-5-((5-pentafluorosulfanyl-2-oxoindolin-3-ylidene)methyl)-1*H*-pyrrol-3-yl)propanoic acid, **13**

5-(Pentafluorosulfanyl)-1,3-dihydro-indol-2-one (106 mg, 0.41 mmol), 3-(5-formyl-1*H*-pyrrole-3-yl)propanoic acid (97.6 mg, 0.5 mmol), ethanol (6 mL) and piperidine (5 drops) were subjected to microwave irradiation by ramping to 150 °C and were held at that temperature for 30 minutes. TLC analysis of the cooled reaction mixture monitored consumption of starting materials. The crude reaction mixture was concentrated, washed with hexane and CH_2_Cl_2_ to give a brown solid. The yield was 141 mg, 79%. ^1^H NMR (DMSO-d_6_, 500 MHz): *δ* = 13.46 (1H, s, OH), 8.40 (1H, s, NH), 7.86 (1H, s, NH), 7.55 (1H, d, *J* = 8.6 Hz, CH), 6.98 (1H, *J* = 8.6 Hz, CH), 2.77–2.72 (2H, m, 2CH), 2.62 (2H, t, *J* = 7.7 Hz, CH_2_), 2.31 (3H, s, CH_3_), 2.28–2.22 (2H, s, CH_2_), 1.48 (3H, s). ^13^C NMR (DMSO-d_6_, 126 MHz): *δ* = 186.1, 174.6, 170.0, 140.1, 136.8, 132.7, 126.7, 126.3, 123.6, 116.2, 110.4, 109.0, 88.3, 88.2, 35.2, 20.0, 12.5, 10.1. HRMS-ESI (*m*/*z*) found: 459.0772, calc. for [C_18_H_17_F_5_N_2_NaO_3_S]^+^ 459.0772. Anal. calcd (%) for C_18_H_17_F_5_N_2_O_3_S: C, 49.54; H, 3.93; N, 6.42. Found (%): C, 49.63; H, 4.04; N, 6.48.

### (*Z*)-3-(2,4-Dimethyl-5-((6-pentafluorosulfanyl-2-oxoindolin-3-ylidene)methyl)-1*H*-pyrrol-3-yl)propanoic acid, **14**

The title compound was prepared by a Knoevenagel condensation reaction. 6-(Pentafluorosulfanyl)1,3-dihydro-indol-2-one (106 mg, 0.41 mmol), 3-(5-formyl-1*H*-pyrrole-3-yl)propanoic acid (97.6 mg, 0.5 mmol), ethanol (6 mL) and piperidine 5 drops were subjected to the microwave irradiation by ramping to 150 °C and were held at that temperature for 30 minutes. TLC analysis of the cooled reaction mixture monitored consumption of starting materials. The crude reaction mixture was dried, washed with hexane and CH_2_Cl_2_ to give a brown solid. The yield was 136 mg, 76%. ^1^H NMR (DMSO-d_6_, 500 MHz): *δ* = 13.50 (1H, s, OH), 10.87 (1H, s, NH), 7.90 (1H, d, *J* = 8.6 Hz, CH), 7.74 (1H, s, NH), 7.46 (1H, dd, *J* = 8.6, 2.1 Hz, CH), 7.24 (1H, d, *J* = 2.1 Hz, CH), 2.78–7.69 (1H, m, CH), 2.66–2.61 (2H, m, CH_2_), 2.34–2.27 (6H, m, 2CH_3_), 2.25 (1H, s, CH), 1.50 (1H, s, CH). ^13^C NMR (DMSO-d_6_, 126 MHz): *δ* = 174.5, 169.7, 137.8, 133.2, 130.4, 126.9, 123.9, 117.9, 109.8, 88.3, 88.2, 44.4, 35.1, 23.1, 22.5, 20.0, 12.5, 9.96. HRMS-ESI (*m*/*z*) found: 459.0776, calc. for [C_18_H_17_F_5_N_2_NaO_3_S]^+^ 459.0772. Anal. calcd (%) for C_18_H_17_F_5_N_2_O_3_S: C, 49.54; H, 3.93; N, 6.42. Found (%): C, 49.70; H, 4.09; N, 6.56.

## Conflicts of interest

There are no conflicts to declare.

## Supplementary Material

Supplementary information

Crystal structure data
