# Correction: Synthesis of kinase inhibitors containing a pentafluorosulfanyl moiety

**DOI:** 10.1039/c7ob90195j

**Published:** 2017-12-19

**Authors:** Supojjanee Sansook, Cory A. Ocasio, Iain J. Day, Graham J. Tizzard, Simon J. Coles, Oleg Fedorov, James M. Bennett, Jonathan M. Elkins, John Spencer

**Affiliations:** Dept of Chemistry, School of Life Sciences, University of Sussex Falmer BN1 9QJ UK j.spencer@sussex.ac.uk; UK National Crystallography Service, Chemistry, University of Southampton Highfield Southampton SO17 1BJ UK; Structural Genomics Consortium, Nuffield Department of Clinical Medicine, University of Oxford Oxford OX3 7DQ UK; Structural Genomics Consortium, Universidade Estadual de Campinas Campinas SP 13083-886 Brazil

## Abstract

Correction for ‘Synthesis of kinase inhibitors containing a pentafluorosulfanyl moiety’ by Supojjanee Sansook *et al.*, *Org. Biomol. Chem.*, 2017, **15**, 8655–8660.

The incorrect CCDC numbers (154150–154153) were quoted in the footnote referencing the electronic supplementary information (ESI) and also in the Experimental section. The correct ESI footnote is shown below with CCDC numbers 1564150–1564153. The CIF files posted originally were also incorrect. These were replaced with the correct files on 29th November 2017.

†Electronic supplementary information (ESI) available. CCDC 1564150–1564153. For ESI and crystallographic data in CIF or other electronic format see DOI: 10.1039/c7ob02289a

The Royal Society of Chemistry apologises for these errors and any consequent inconvenience to authors and readers.

## Supplementary Material

