# Supplementary material for: Synthesis of kinase inhibitors containing a pentafluorosulfanyl moiety
Source: Org Biomol Chem. 2017 Sep 29;15(40):8655–60. doi: 10.1039/c7ob02289a (PMC5708334; doi:10.1039/c7ob02289a)
Supplement: Supplementary file 1 [file OB-015-C7OB02289A-s001.pdf]

Electronic Supplementary Material (ESI) for Organic & Biomolecular Chemistry.
This journal is © The Royal Society of Chemistry 2017

Synthesis of kinase inhibitors containing a pentafluorosulfanyl moiety.

Supojjanee Sansook[a], Cory A. Ocasio[a], Iain J. Day[a], Graham J. Tizzard[b], Simon J. Coles[b], Oleg Fedorov[c], James M. Bennett[c], Jonathan M. Elkins[c,d], John Spencer[a,*]

[a]*Dept of Chemistry, School of Life Sciences, University of Sussex, Falmer, BN1 9QJ, UK.* [b]UK National Crystallography Service, Chemistry, University of Southampton, Highfield, Southampton, SO17 1BJ, U.K. [c]Structural Genomics Consortium, Nuffield Department of Clinical Medicine, University of Oxford, Oxford, OX3 7DQ, UK. [d]Structural Genomics Consortium, Universidade Estadual de Campinas, Campinas, SP 13083-886, Brazil [e]Structural Genomics Consortium, Universidade Estadual de Campinas, Campinas, SP 13083-886, Brazil.

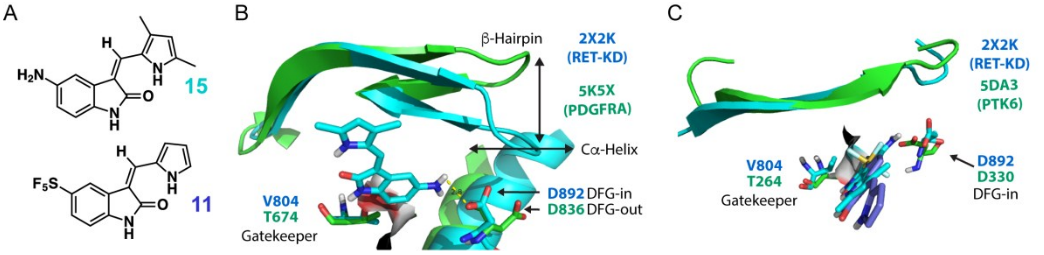

Figure S1. Structural studies comparing binding modes of methylidene indolinone-based kinase inhibitors **11** and **15** (A) with active and inactive kinase domains. Compound **15** (A) was co-crystallised with RET kinase domain (B, PDB: 2X2K) forcing a "DFG-in" kinase conformation. This **15**-bound RET conformation was aligned with the PDGFRA crystal structure (B, PDB: 5K5X) revealing gross conformational shifts around the ATP-binding pocket, particularly between the RET and PDGFRA b-hairpin and Ca-helix (B), and compared to RET, the PDGFRA DFG catalytic-motif aspartic acid is pointing outside of the ATP-binding pocket; evidence for an inactive kinase conformation. Compound **11** displayed the greatest potency in the series against PDGFRA, a receptor tyrosine kinase containing a threonine gatekeeper. RET has a valine gatekeeper, and thus PTK6 (C, PDB: 5DA3), a non-receptor tyrosine kinase containing a threonine gatekeeper, was selected for docking studies with compound **10** - **11** (Fig. 5, main article) to ascertain the molecular determinants for the superior potency of **11** vs **10**. The alignment between 15-bound RET and **11**-docked PTK6 (C) reveals very similar binding modes between compounds **11** and **15** and an agreement in PTK6 and RET kinase conformation, both in the active state.

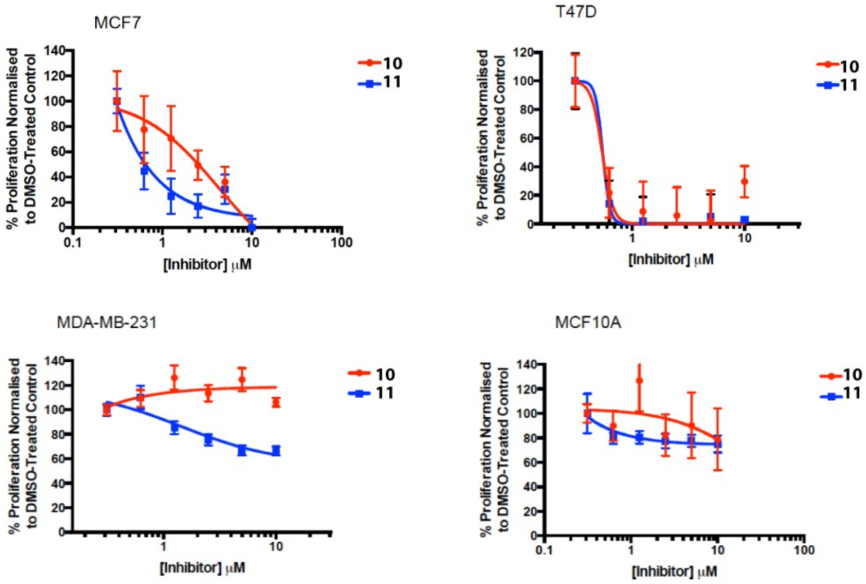

Figure S2. Proliferation assays. Dose-dependent inhibition of MCF7, T47D MDA-BM-231 and MCF10A cells by compounds **10** and **11**.

Fig S3. NMRs

(*Z*)-3-((1H-Pyrrol-2-yl)methylene-5-pentafluorosulfanylindoline-2-one

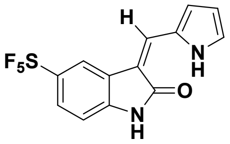

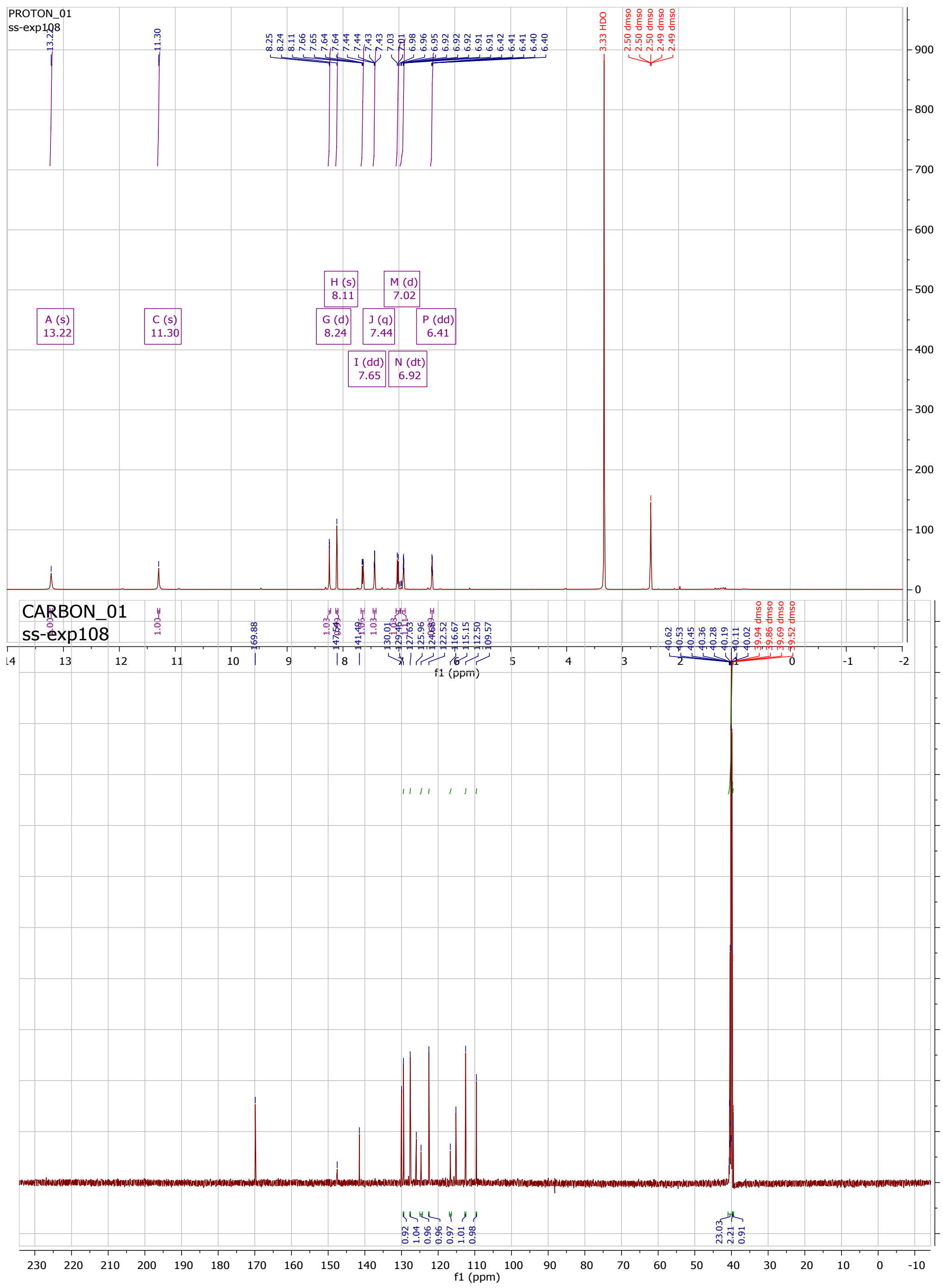

(*Z*)-3-((1H-Pyrrol-2-yl)methylene-6-pentafluorosulfanylindoline-2-one

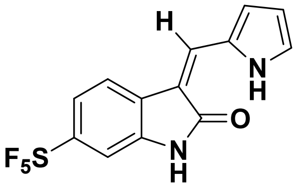

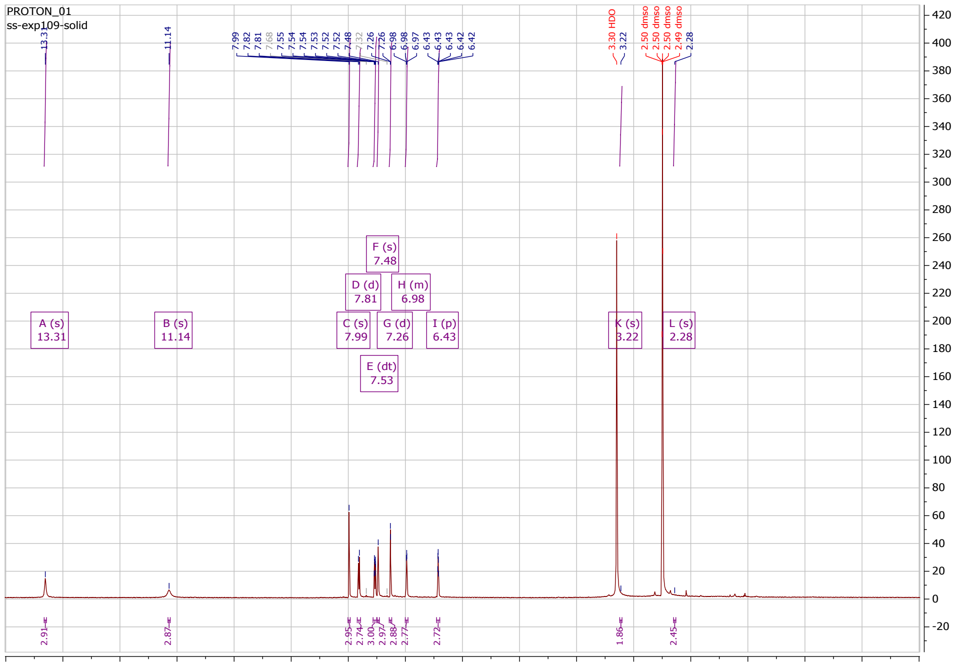

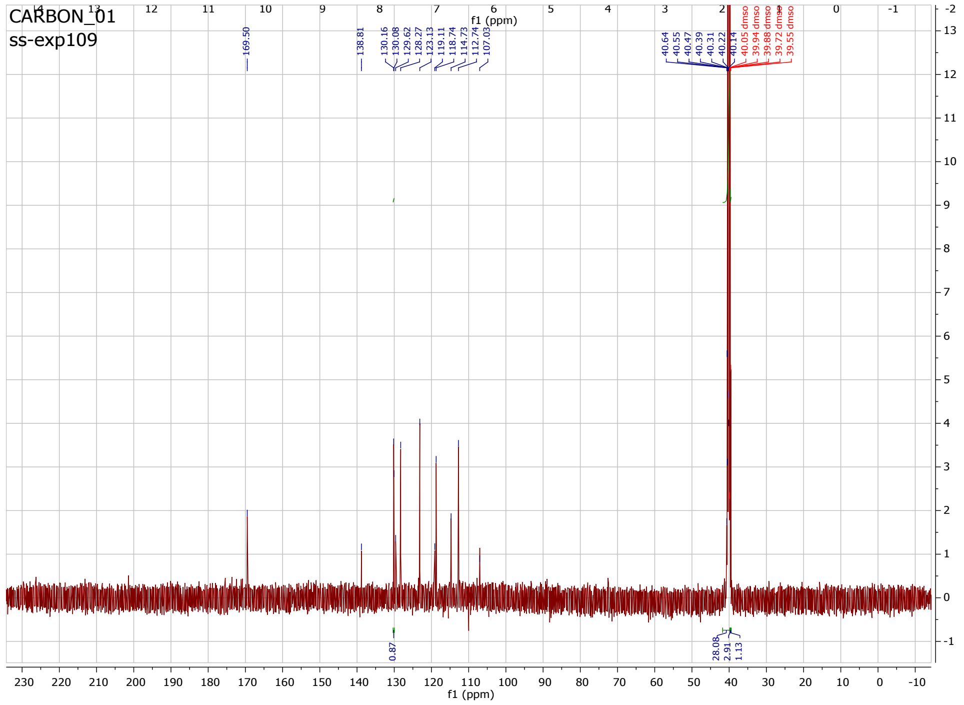

(*E*)-5-Pentafluorosulfanyl-3-ferrocenylindolin-2-one

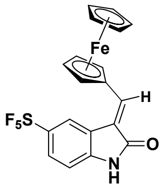

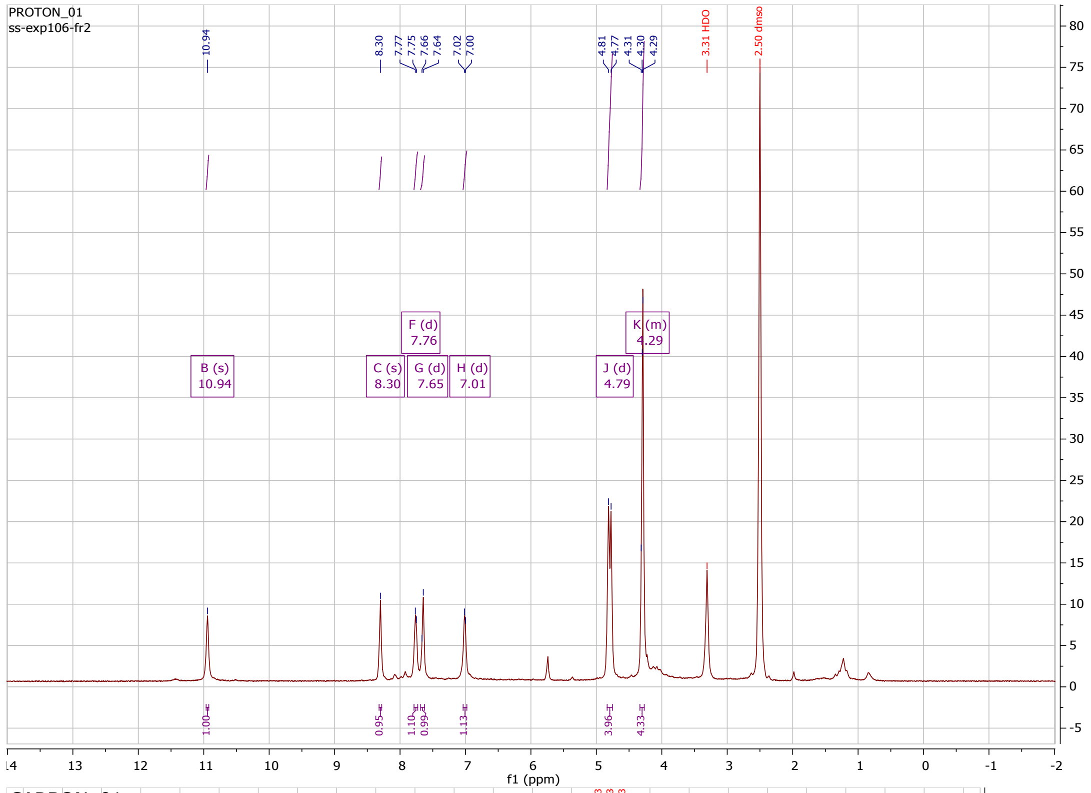

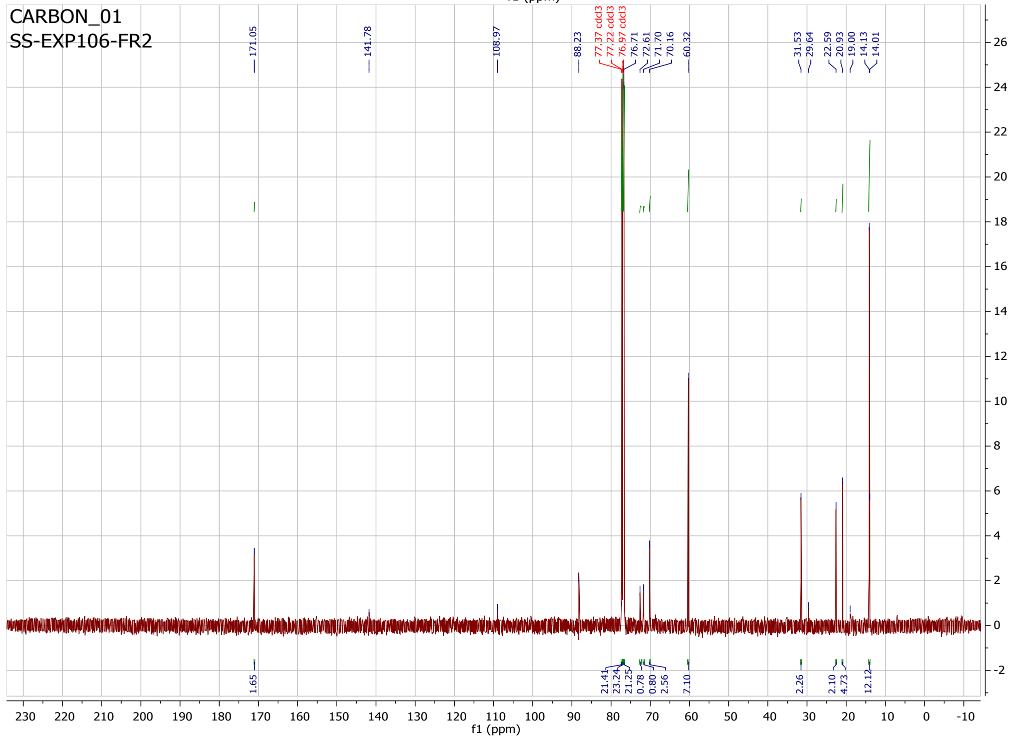

(*Z*)-5-Pentafluorosulfanyl-3-ferrocenylindolin-2-one

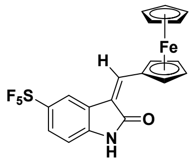

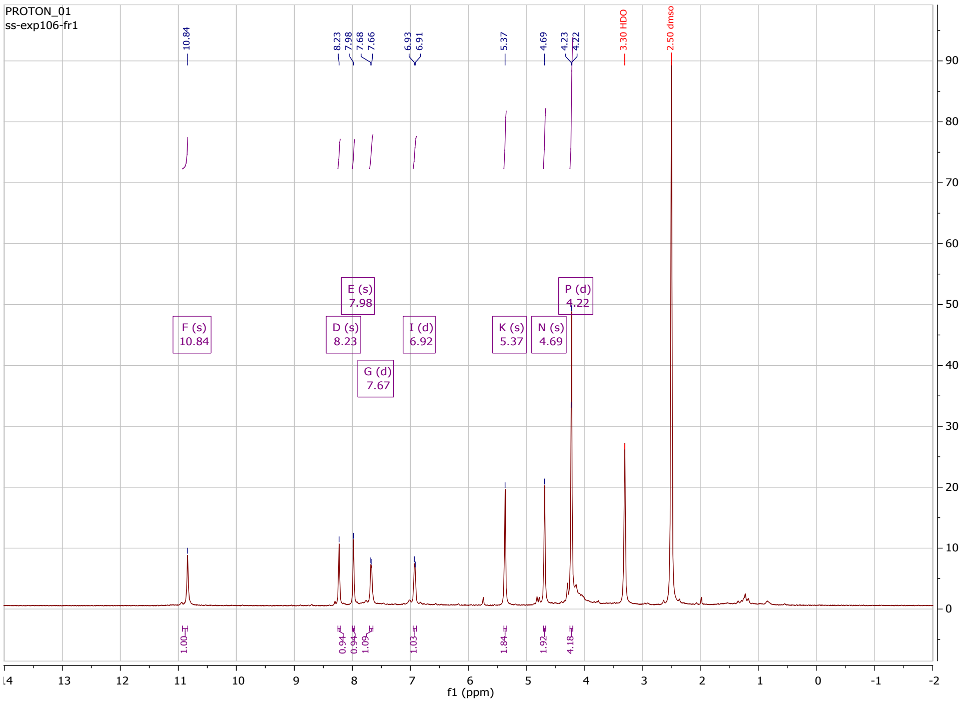

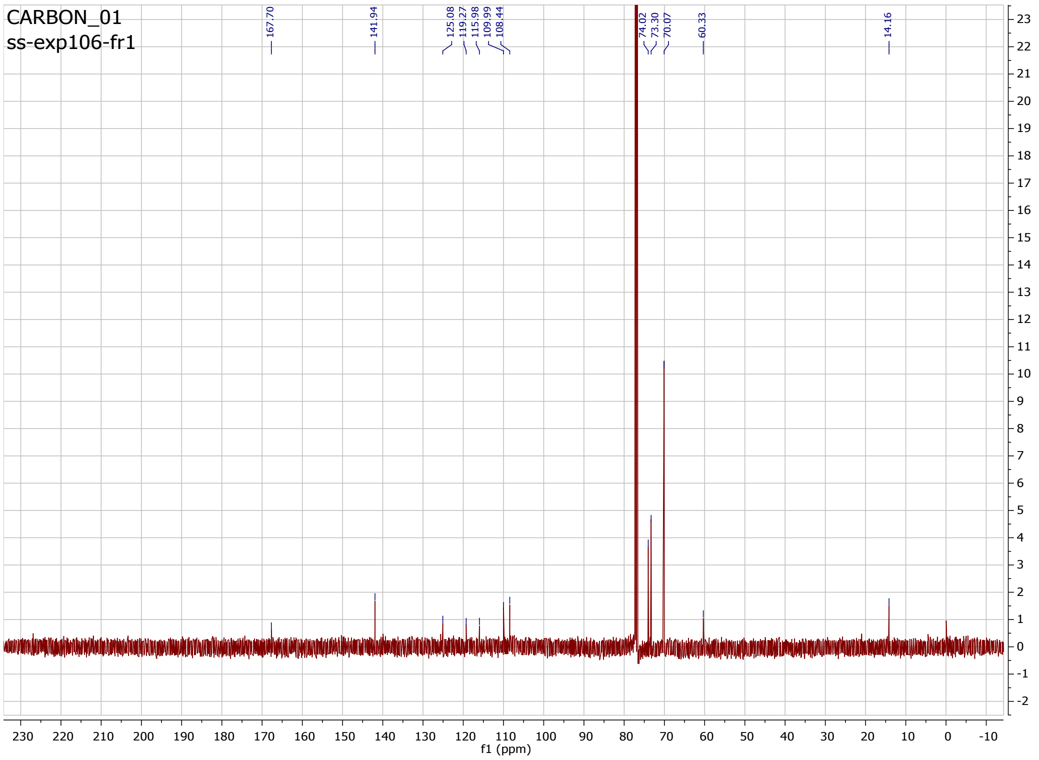

(*Z*)-3-(2,4-dimethyl-5-((5-pentafluorosulfanyl-2-oxoindolin-3-ylidene)methyl)-1H-pyrrol-3-yl)propanoic acid

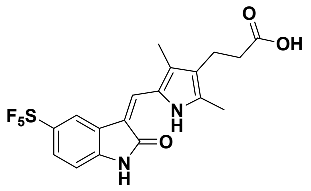

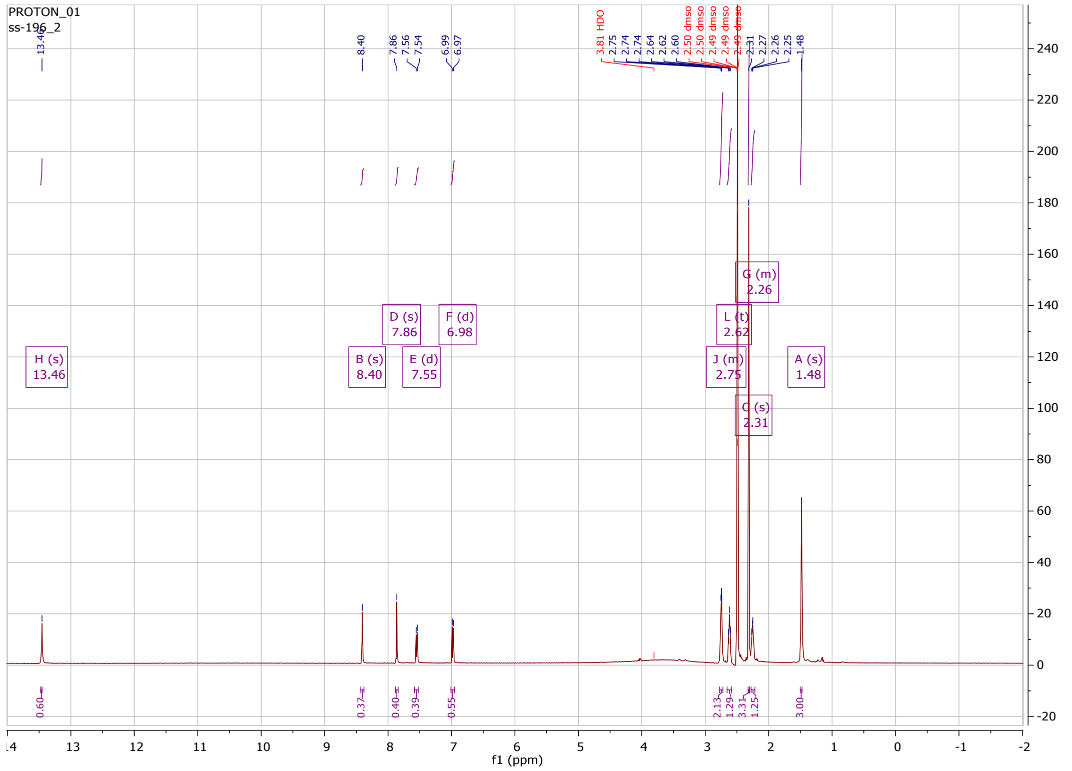

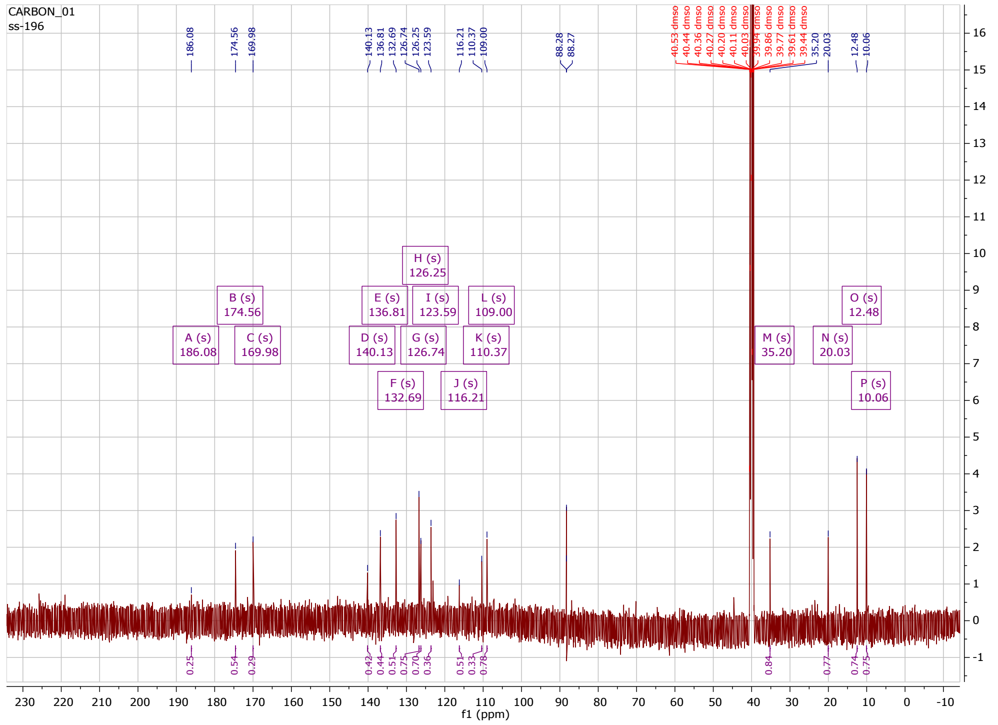

(*Z*)-3-(2,4-dimethyl-5-((6-pentafluorosulfanyl-2-oxoindolin-3-ylidene)methyl)-1H-pyrrol-3-yl)propanoic acid

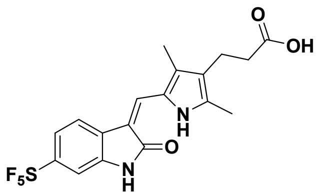
O
OH
N
H
O
N
H
F5S

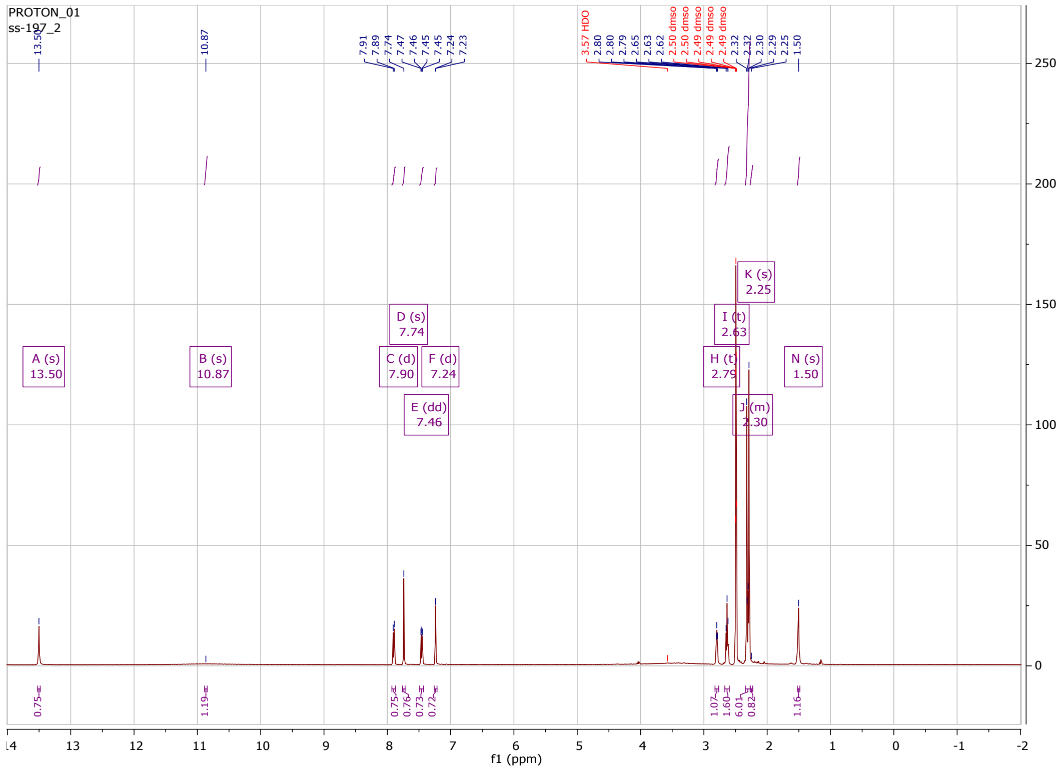
PROTON_01
ss-197_2
13.50
10.87
7.91
7.89
7.74
7.47
7.46
7.45
7.45
7.24
7.23
3.57 HDO
2.80
2.80
2.79
2.65
2.63
2.62
2.50 dmso
2.50 dmso
2.49 dmso
2.49 dmso
2.49 dmso
2.32
2.32
2.30
2.29
2.25
1.50
A (s)
13.50
B (s)
10.87
C (d)
7.90
D (s)
7.74
E (dd)
7.46
F (d)
7.24
H (t)
2.79
I (t)
2.63
J (m)
2.30
K (s)
2.25
N (s)
1.50
0.75
1.19
0.75
0.76
0.73
0.72
1.07
1.60
6.01
0.82
1.16
f1 (ppm)

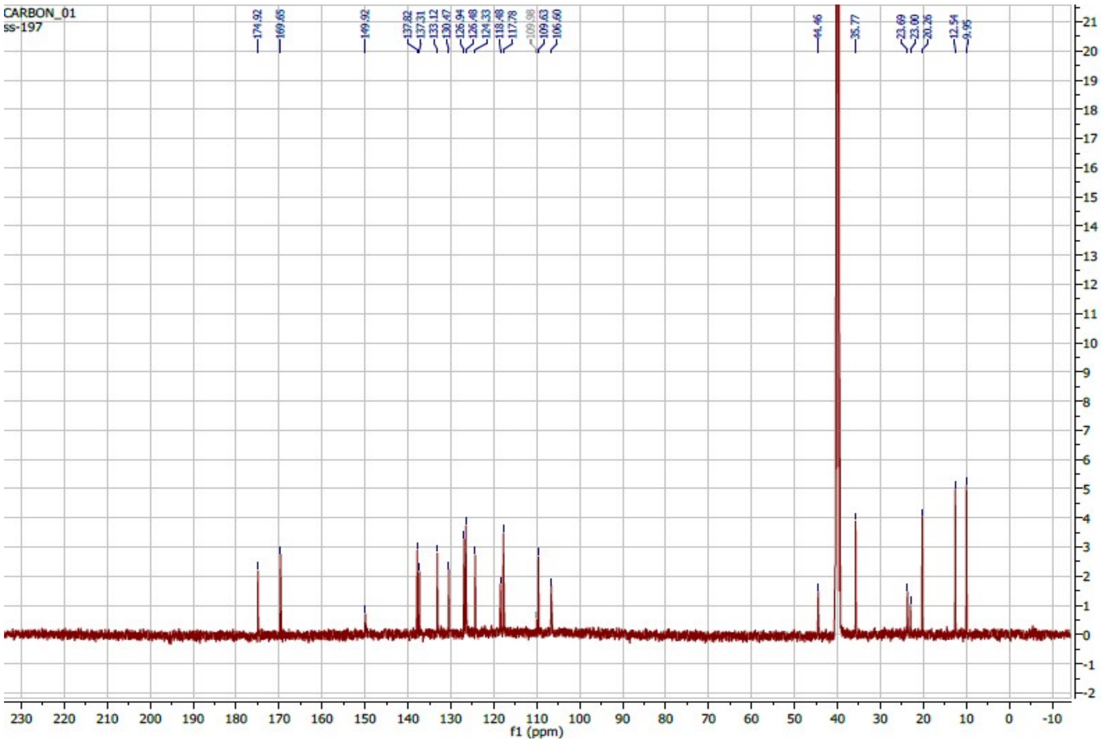
CARBON_01
ss-197
174.92
169.65
149.92
137.82
137.31
133.12
130.47
126.94
126.48
124.33
118.48
117.78
109.98
109.63
106.60
44.46
35.77
23.69
23.00
20.26
12.54
9.95
f1 (ppm)

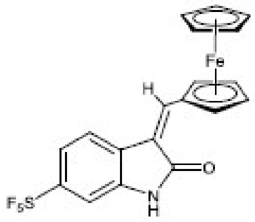
Fe
H
O
N
H
F5S

[19]F: (broad)

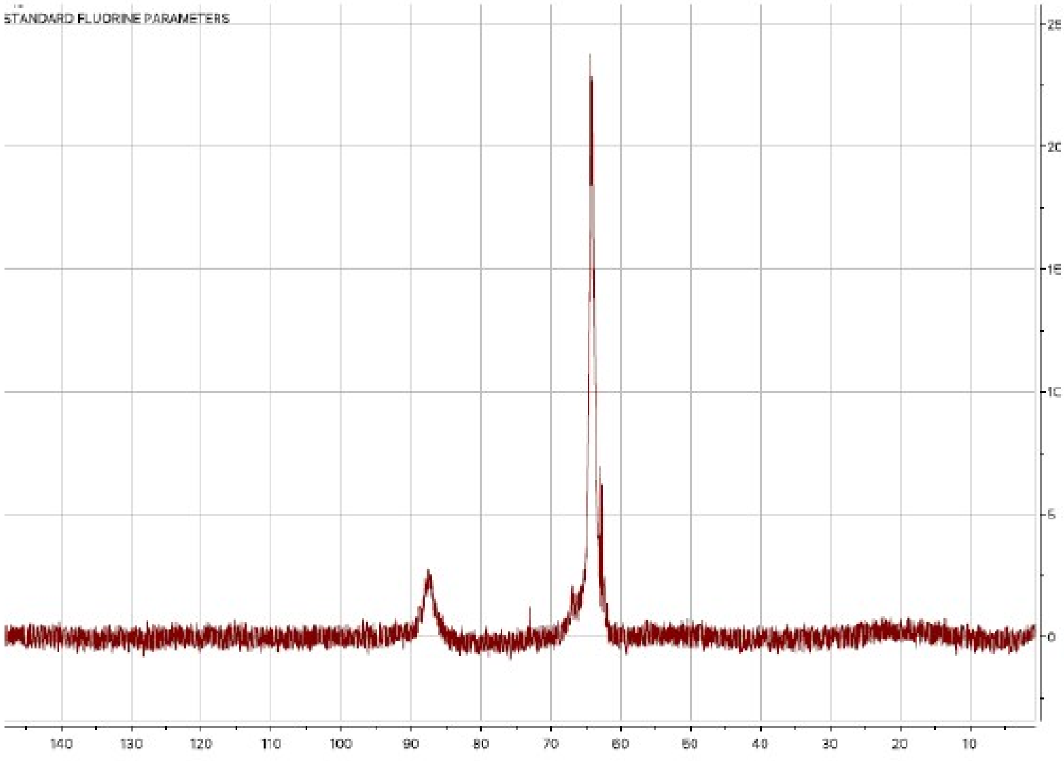
STANDARD FLUORINE PARAMETERS
140
130
120
110
100
90
80
70
60
50
40
30
20
10
0
5

^{19}F

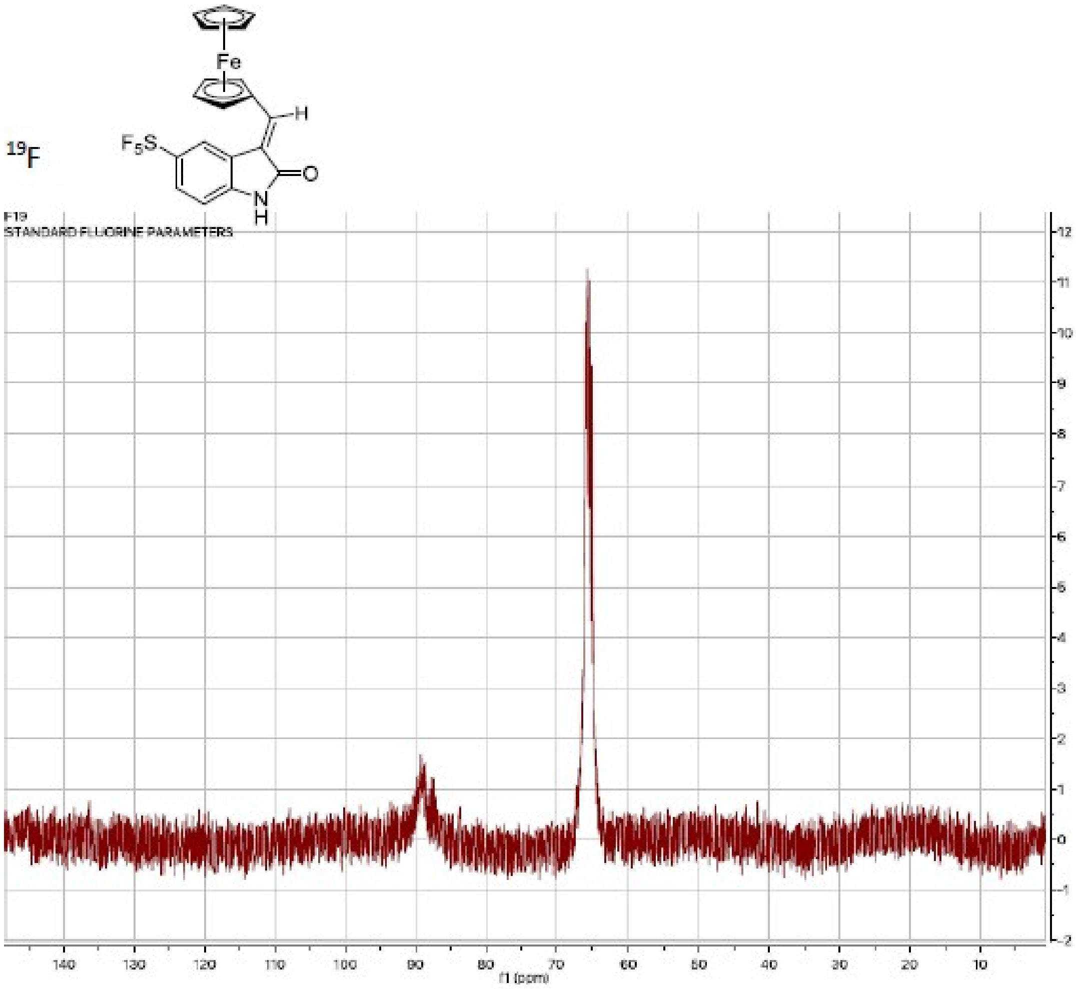

^{19}F

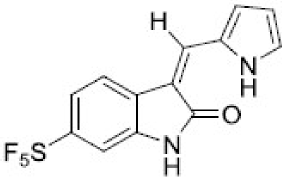

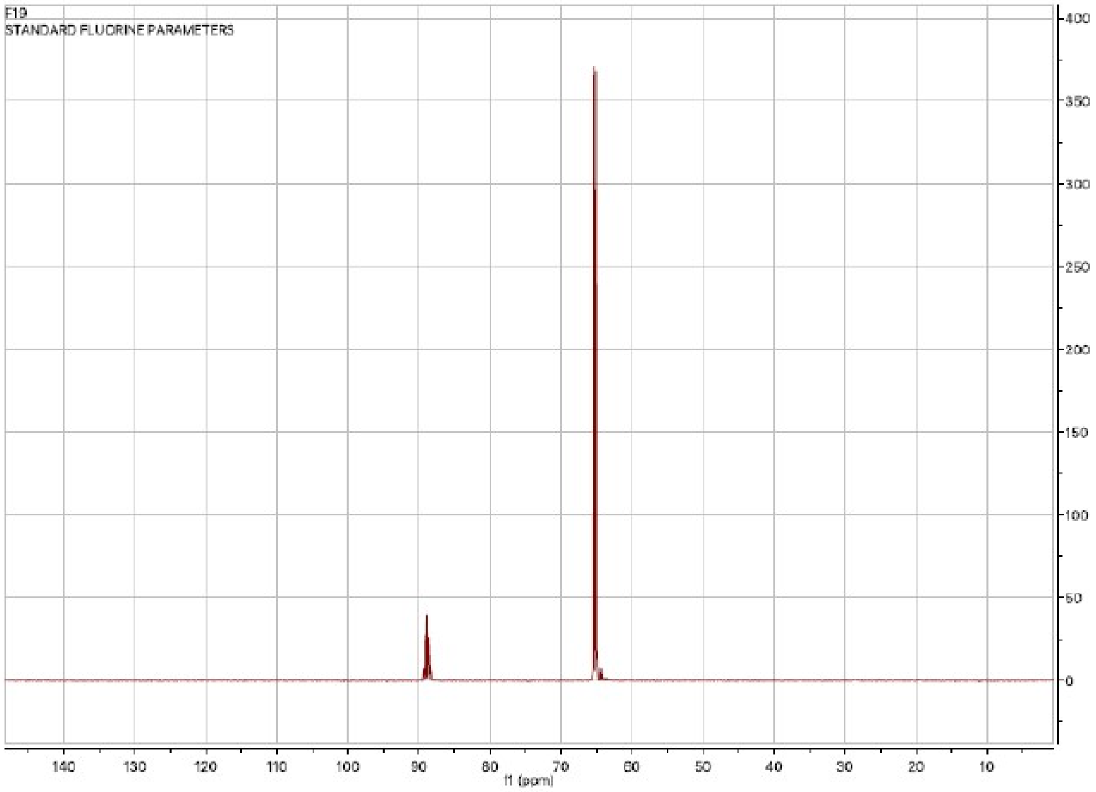

^{19}F

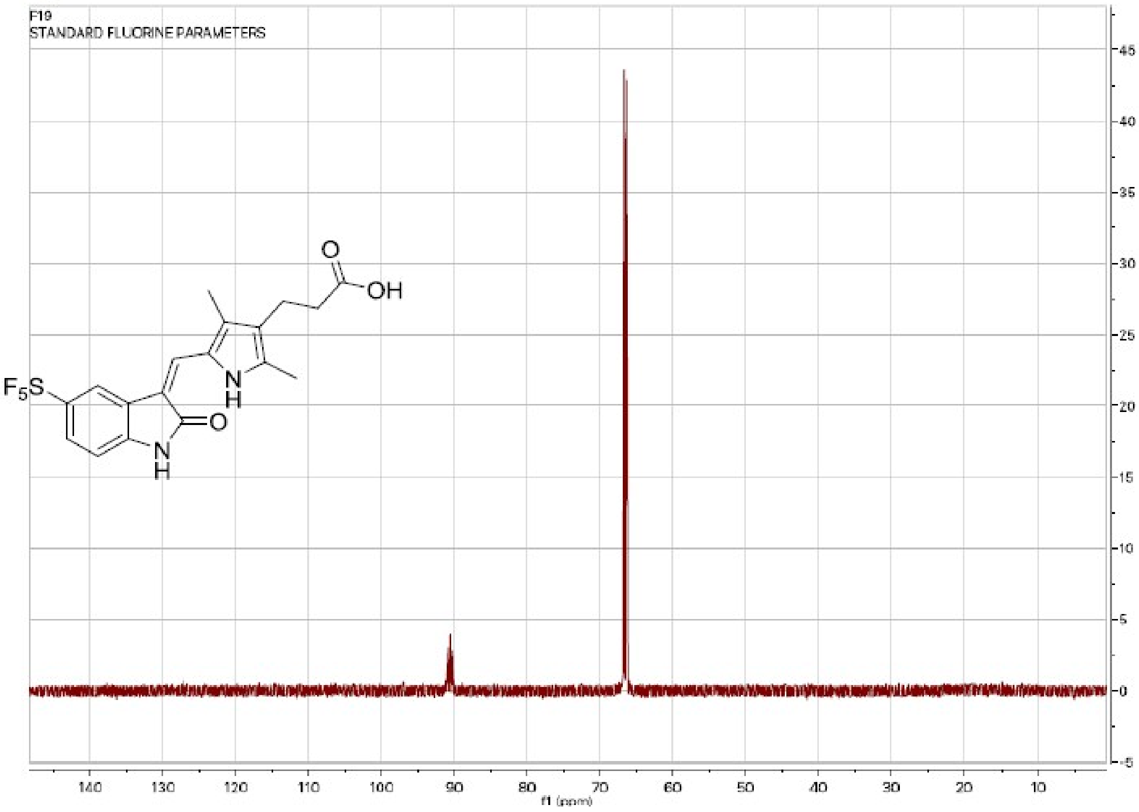
F19
STANDARD FLUORINE PARAMETERS
F5S
OH
O
N
H
f1 (ppm)

^{19}F

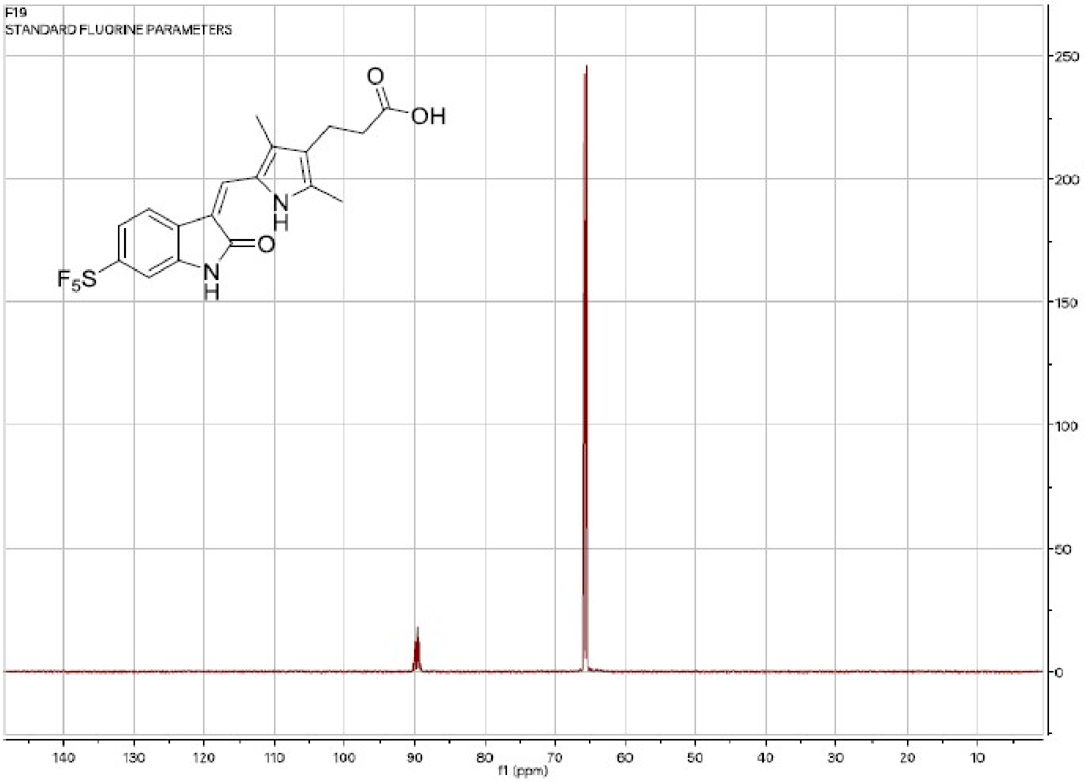
F19
STANDARD FLUORINE PARAMETERS
F5S
OH
O
N
H
f1 (ppm)
